# Nonstationary Temperature-Duration-Frequency curves

**DOI:** 10.1038/s41598-018-33974-y

**Published:** 2018-10-19

**Authors:** Taha B. M. J. Ouarda, Christian Charron

**Affiliations:** Canada Research Chair in Statistical Hydro-Climatology, INRS-ETE, 490 de la Couronne, Québec, QC G1K 9A9 Canada

## Abstract

Persistent extreme heat events are of growing concern in a climate change context. An increase in the intensity, frequency and duration of heat waves is observed in several regions. Temperature extremes are also influenced by global-scale modes of climate variability. Temperature-Duration-Frequency (TDF) curves, which relate the intensity of heat events of different durations to their frequencies, can be useful tools for the analysis of heat extremes. To account for climate external forcings, we develop a nonstationary approach to the TDF curves by introducing indices that account for the temporal trend and teleconnections. Nonstationary TDF modeling can find applications in adaptive management in the fields of health care, public safety and energy production. We present a one-step method, based on the maximization of the composite likelihood of observed heat extremes, to build the nonstationary TDF curves. We show the importance of integrating the information concerning climate change and climate oscillations. In an application to the province of Quebec, Canada, the influence of Atlantic Multidecadal Oscillations (AMO) on heat events is shown to be more important than the temporal trend.

## Introduction

Extended periods of extreme temperature have significant adverse impacts on public health, infrastructure and natural ecosystems. It is well established that mean temperatures have increased globally since the middle of the 20th century^1^.This phenomenon has been attributed to the human influence on climate^2^. Increase in global mean temperature has resulted in an even larger increase in the probabilities of extreme temperature events^3–5^. Recently, a large number of heat waves occurred in different regions^6,7^, causing considerable damage. It is expected that the frequency, duration and intensity of extreme heat events will increase in a future warmer climate^8–10^. Aside from climate change, global-scale modes of climate variability have also important influences on temperature extremes around the world. The links between temperature extremes and circulation patterns have been demonstrated in a number of studies^11–13^.

Rainfall Intensity-Duration-Frequency (IDF) curves are widely used tools for the planning, design and operation of water resources infrastructure^14,15^. IDF curves relate rainfall intensities corresponding to different durations to a series of return periods. A similar concept applied to temperature extremes^16^, referred to in this work as the Temperature-Duration-Frequency (TDF) curves approach, would be of interest for managers in a number of fields including healthcare, public security, agriculture and energy production.

In the only previous work on TDF curves, it was assumed that the characteristics of the probability distribution of extreme heat events are invariant through time^16^. However, temperature extreme characteristics change with time due to the influence of climate change and climate oscillation patterns. In this context, it is important to develop models for temperature extremes that account for external forcings. One approach to handle nonstationarity in temperature extremes is to make the parameters of the temperature extremes distribution dependent on covariates representing climate variability and change^17^. A covariate representing time can be used to account for the eventual temporal trend caused by climate change, and covariates representing climate indices can be introduced to take into consideration the influence of climate oscillation patterns.

The mathematical formulation of the non-stationary TDF curves presented here follows the one of the general IDF relationship (see Methods) but extended to the non-stationary case. In this formulation, a single model is defined in which the duration is a parameter. This model differs from other recent works introducing non-stationarity in IDF curves where an independent model is defined for each duration. Maximum temperatures are modeled with the Generalized Extreme Value (GEV) distribution, a 3-parameter distribution widely used to model climatic extremes.

Such a nonstationary tool would be particularly useful in adaptive management, a structure process of decision making in condition of uncertainty^18^. This concept has been used, for instance, in the fields of agriculture^19^, health care^20^ and water resources^21,22^. Adaptive management is expected to become an important tool for public health policy makers where knowledge concerning trends in climate extremes can be used for mitigation and preparedness^23^. Non stationary TDF curves can also be used to integrate information concerning future temperature extreme characteristics in the design, operation, and safety analysis of thermal and nuclear power plants^24^.

## Results

### Modelling of extreme temperature with nonstationary TDF curves

The nonstationary TDF approach proposed here is applied to observed daily maximum temperatures at meteorological stations in the Province of Quebec (Canada). Six meteorological stations with long series and recent data were selected. Table 1 presents the selected stations with their coordinates and the parameters obtained for the stationary TDF approach. To build the TDF curves, the annual maximum of multi-day averages of daily maximum temperatures over durations of 1 to 7 and 10 days (a total of 8 durations) during the summer season (June-August) were extracted for each station. The slope of the annual maximum temperature time series was computed for each duration using the Theil-Sen method. The slopes corresponding to all durations have a positive sign at all stations except for the station of Quebec Intl. Airport where the slopes are negative for all durations (Supplementary Table 1). An analysis of the trends with the non-parametric Mann-Kendall statistical test reveals that, while trends are not generally significant, positive significant trends are observed for most of the durations for the stations of Sherbrooke and Rimouski.

**Table 1:** Description of selected stations and parameters of the stationary TDF curves.

Station	Latitude	Longitude	Period	*μ*	*σ*	*κ*	*θ*	*η*
Montréal Intl. Airport	45.47	−73.74	1941–2015	35.48	1.71	−0.22	1.94	0.09
Quebec Intl. Airport	46.80	−71.38	1943–2016	36.52	1.83	−0.23	2.77	0.11
Sherbrooke	45.44	−71.69	1962–2015	36.88	1.68	−0.35	3.54	0.12
Rimouski	48.45	−68.52	1953–2016	33.54	1.97	−0.30	1.05	0.11
Bagotville	48.33	−71.00	1943–2016	36.26	2.12	−0.31	1.35	0.12
Ste-Anne-de-la-Pérade	46.58	−72.23	1950–2016	35.03	1.76	−0.17	1.99	0.10

The Atlantic Multidecadal Oscillation (AMO) is a large scale pattern of multidecadal variability related to variations of the sea surface temperature in the North Atlantic Ocean. Several examples of regional multidecadal climate variability have been related to the AMO including for the North American and European summer climate^25^. AMO is known to have the most important influence on summer temperature extremes in North America^26^ and is selected here to be included as covariate in the nonstationary TDF approach. Annual AMO time series for the summer season are obtained by averaging the monthly values over the summer months (June-August) of each year. Correlations of summer AMO with the annual maximum temperatures for the different durations at the six stations are computed and the Student’s *t*-test reveals that correlations are significant at a significance level of 5% for most of the durations (Supplementary Table 2).

In the nonstationary framework, we consider the location and scale parameters of the GEV distribution in the TDF relationship to be dependent upon the covariates. The variables “Time” representing the year, and the climate index AMO for the summer season, are used as covariates (see Methods). One nonstationary model uses Time as covariate (denoted TDF model “Time”), a second uses AMO (denoted TDF model “AMO”) and a third model uses both covariates together (denoted TDF model “Time + AMO”). The parameters of the TDF relationships are estimated with the maximum composite likelihood method (see Methods for details). The criterion CL-AIC (see equation (14) in Methods), an analogue of the Akaike information criterion (AIC), is used for model comparison.

Table 2 presents the maximized independence log-likelihood, the CL-AIC statistic and the model parameters for each TDF model and each station. For a given station and a given selection of covariates, the table presents only the best model among the linear and quadratic relationships presented in equations (6–9) according to the CL-AIC statistic. Table 2 shows that the linear relationship is selected by CL-AIC in all cases except in the case of Montréal where a quadratic relationship is selected when the covariate is time. In all cases, with respect to the CL-AIC criterion, the goodness-of-fit is improved when a nonstationary model is used. For the majority of stations, the model with only AMO obtains the best CL-AIC values. This means, that for most stations, the influence of the climate oscillation pattern is more important than the temporal trend. This result is in agreement with the fact that correlations with the AMO index are more significant than temporal trends. For the Quebec and the Rimouski stations, the best goodness-of-fit is obtained with a combination of covariates Time and AMO. This indicates that in some cases the combination of the two covariates has significant impacts on extreme temperatures.

**Table 2:** Statistics and model parameters for the stationary and nonstationary TDF curves.

Station	Model	${{\boldsymbol{\ell }}}_{{\boldsymbol{ind}}}$	CL-AIC	Model parameters
Montréal Intl. Airport	Stationary	−1084.2	2205.5	*μ*, *σ*, *κ*, *θ*, *η*
	Time	−1068.0	2200.7	*μ*_*l*_ = *μ*_0_ + *μ*_1_*Time* + *μ*_2_*Time*^2^, *σ*, *κ*, *θ*, *η*
	AMO	−1064.7	**2179.7**	*μ*_*l*_ = *μ*_0_ + *μ*_1_*AMO*, *σ*, *κ*, *θ*, *η*
	Time + AMO	**−1064.7**	2194.3	*μ*_*l*_ = *μ*_0_ + *μ*_1_*Time* + *μ*_2_*AMO*, *σ*, *κ*, *θ*, *η*
Quebec Intl. Airport	Stationary	−1083.7	2203.1	*μ*, *σ*, *κ*, *θ*, *η*
	Time	−1074.7	2197.2	*μ*, *σ*_*l*_ = *σ*_0_ + *σ*_1_*Time*, *κ*, *θ*, *η*
	AMO	−1068.7	2187.8	*μ*_*l*_ = *μ*_0_ + *μ*_1_*AMO*, *σ*, *κ*, *θ*, *η*
	Time + AMO	**−1046.3**	**2175.8**	*μ*_*l*_ = *μ*_0_ + *μ*_1_*Time* + *μ*_2_*AMO*, *σ*_*l*_ = *σ*_0_ + *σ*_1_*Time* + *σ*_2_*AMO*, *κ*, *θ*, *η*
Sherbrooke	Stationary	−704.9	1443.4	*μ*, *σ*, *κ*, *θ*, *η*
	Time	−686.2	1429.6	*μ*_*l*_ = *μ*_0_ + *μ*_1_*Time*, *σ*_*l*_ = *σ*_0_ + *σ*_1_*Time*, *κ*, *θ*, *η*
	AMO	−674.2	**1394.8**	*μ*_*l*_ = *μ*_0_ + *μ*_1_*AMO*, *σ*, *κ*, *θ*, *η*
	Time + AMO	**−663.8**	1404.7	*μ*_*l*_ = *μ*_0_ + *μ*_1_*Time* + *μ*_2_*AMO*, *σ*_*l*_ = *σ*_0_ + *σ*_1_*Time* + *σ*_2_*AMO*, *κ*, *θ*, *η*
Rimouski	Stationary	−912.7	1856.0	*μ*, *σ*, *κ*, *θ*, *η*
	Time	−884.2	1815.6	*μ*_*l*_ = *μ*_0_ + *μ*_1_*Time*, *σ*_*l*_ = *σ*_0_ + *σ*_1_*Time*, *κ*, *θ*, *η*
	AMO	−893.4	1829.2	*μ*_*l*_ = *μ*_0_ + *μ*_1_*AMO*, *σ*, *κ*, *θ*, *η*
	Time + AMO	**−872.2**	**1810.4**	*μ*_*l*_ = *μ*_0_ + *μ*_1_*Time* + *μ*_2_*AMO*, *σ*_*l*_ = *σ*_0_ + *σ*_1_*Time* + *σ*_2_*AMO*, *κ*, *θ*, *η*
Bagotville	Stationary	−1139.6	2309.1	*μ*, *σ*, *κ*, *θ*, *η*
	Time	−1139.5	2317.4	*μ*, *σ*_*l*_ = *σ*_0_ + *σ*_1_*Time*, *κ*, *θ*, *η*
	AMO	−1123.8	**2289.6**	*μ*_*l*_ = *μ*_0_ + *μ*_1_*AMO*, *σ*, *κ*, *θ*, *η*
	Time + AMO	**−1123.8**	2302.8	*μ*_*l*_ = *μ*_0_ + *μ*_1_*Time* + *μ*_2_*AMO*, *σ*, *κ*, *θ*, *η*
Ste-Anne-de-la-Pérade	Stationary	−988.6	2016.7	*μ*, *σ*, *κ*, *θ*, *η*
	Time	−984.6	2021.4	*μ*, *σ*_*l*_ = *σ*_0_ + *σ*_1_*Time*, *κ*, *θ*, *η*
	AMO	−959.5	**1971.2**	*μ*_*l*_ = *μ*_0_ + *μ*_1_*AMO*, *σ*, *κ*, *θ*, *η*
	Time + AMO	**−958.3**	1982.9	*μ*_*l*_ = *μ*_0_ + *μ*_1_*Time* + *μ*_2_*AMO*, *σ*, *κ*, *θ*, *η*

**Figure 1:**
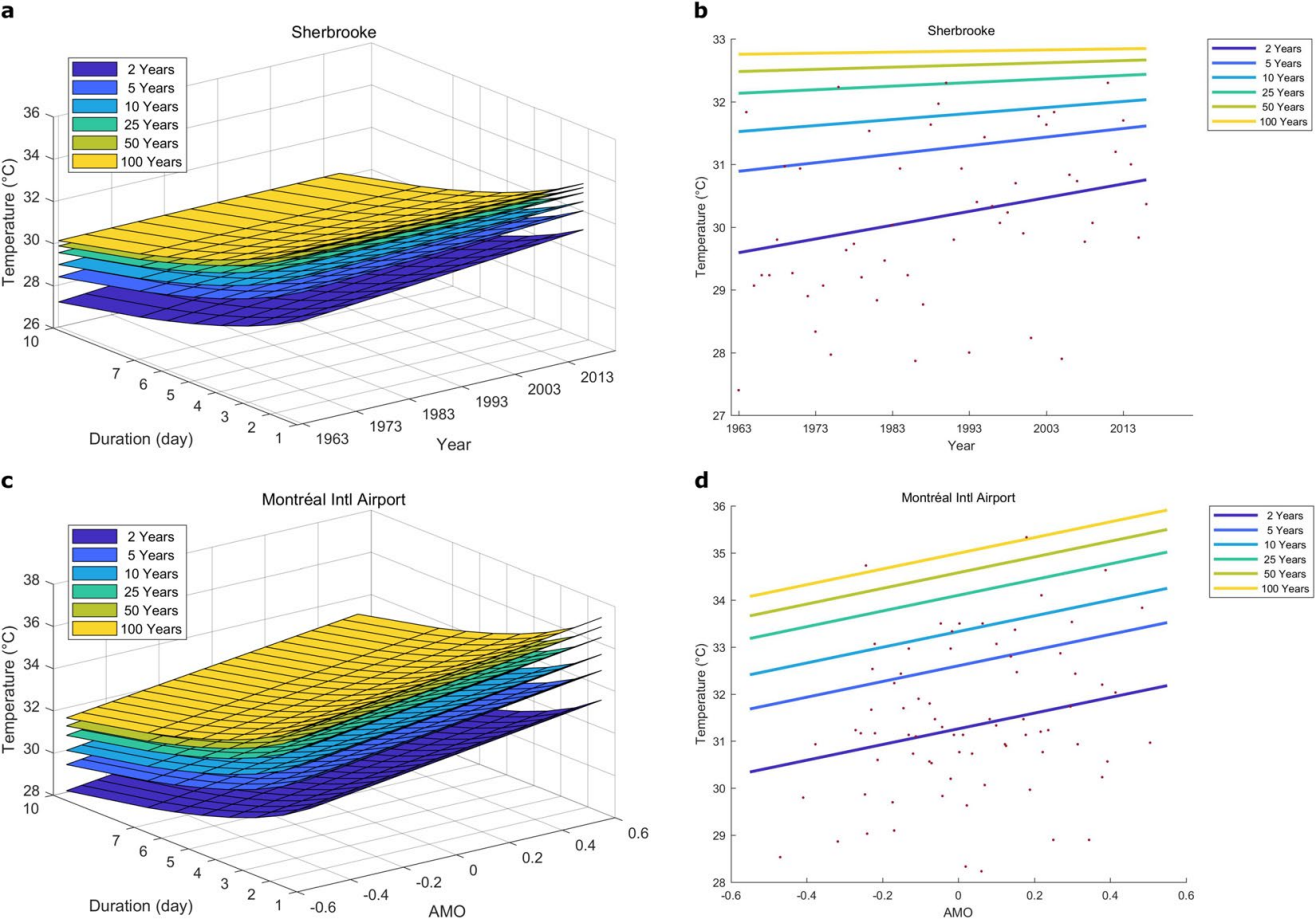
Nonstationary TDF surfaces and 3-day maximum temperatures against the covariate at Sherbrooke and Montréal stations. Nonstationary TDF surfaces are presented for the stations at Sherbrooke (**a**) and Montréal (**c**) with the covariates Time and AMO respectively. The 3-day maximum temperatures are represented for the stations of Sherbrooke (**b**) and Montréal (**d**) against the covariates Time and AMO respectively. Red dots represent observations. (**b**,**d**) are cross sections in (**a**,**c**) respectively.

Stationary TDF curves and similar relationships (e.g. IDF curves) are generally represented on graphs with the intensity plotted against the duration where each curve represents a return period. Such representation is not possible with nonstationary TDF curves. With one covariate, TDF surfaces can be well defined and it is possible to represent them with 3D graphs of the intensity against the duration and the covariate. Two examples of such graphs are presented in Fig. 1a for Sherbrooke with the TDF model “Time” and in Fig. 1c for Montréal with the TDF model “AMO”. Curves of the 3-day maximum temperatures against the covariate are also illustrated for the same examples in Fig. 1b–d. These curves represent cross sections of the TDF surfaces for fixed durations. A positive temporal trend with a decreasing variance can be observed for Sherbrooke and a linear positive relationship with AMO can be observed for Montréal. For Sherbrooke, the location and the scale parameters are related linearly to the covariate Time, and for Montréal, the location parameter is related linearly to the covariate AMO. With two covariates, it is possible to represent TDF surfaces with 3D graphs of the intensity against each covariate for separate durations or separate return periods. Examples of such illustrations are presented in Supplementary Fig. 10, where the 3-day maximum temperatures are plotted against Time and AMO for each station.

### Impacts of considering nonstationarity

**Figure 2:**
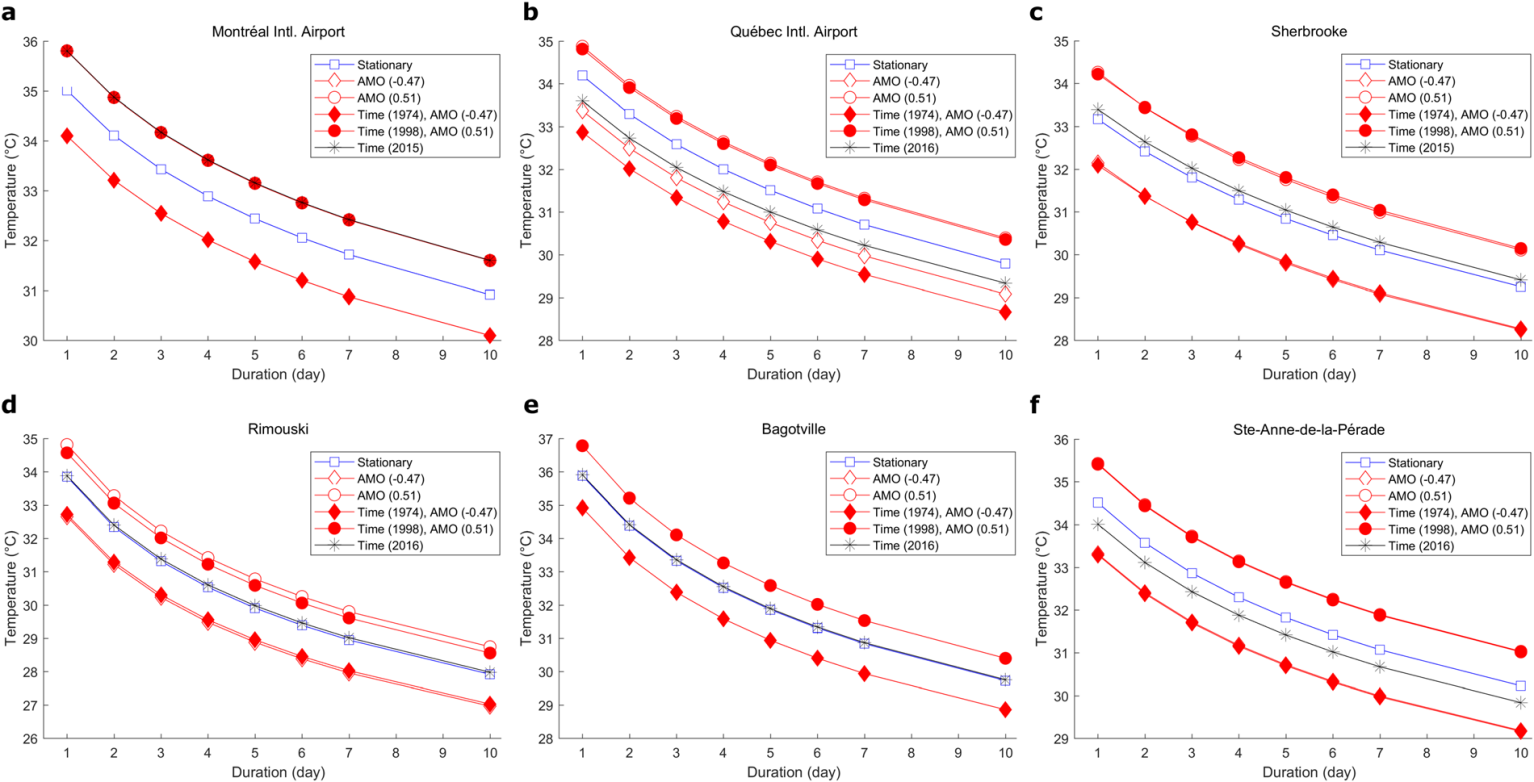
Comparison of the 10-year quantiles for the stationary TDF model and the nonstationary TDF models. 10-year quantiles for the stationary TDF model, the nonstationary TDF model Time for the case of the last year of record, the nonstationary TDF model AMO for the largest and lowest observed values of AMO (−0.47 and 0.51) and the nonstationary TDF model Time + AMO for the 2 years with the largest and lowest observed values of AMO (1974 and 1998) and their corresponding values.

To illustrate the impacts of considering nonstationary TDF curves instead of the classical stationary model, Fig. 2 presents, for each station, graphs of the 10-year quantiles versus the duration for the stationary TDF model and the nonstationay TDF models for values of the covariates representing different scenarios. For the nonstationary TDF model with Time as covariate, the case for the last year of record is considered. This model represents the most probable TDF relationship for the last year of record when only the temporal trend is considered. For the nonstationary TDF models “AMO” and “Time + AMO”, the years with the largest and lowest values of AMO during the period 1941–2016 are identified to illustrate extreme cases (1974 and 1998 for which AMO was respectively at the values −0.47 and 0.51). For the nonstationary TDF model “AMO”, the two observed extreme values of AMO during these two years were considered. Finally, for the nonstationary TDF model “Time + AMO”, these two years and their corresponding AMO values were considered.

Figure 2 illustrates the importance of considering climate variability when building TDF curves, as large discrepancies in quantiles are obtained for different years and for different nonstationary models. The mean difference between the quantiles of the two years selected for the TDF model “Time + AMO” is about 1.8 °C. In general, the climate index has much more impact than the temporal trend on the quantiles. For instance, quantiles obtained with the TDF model “Time” at the Sherbrooke, Rimouski and Bagotville stations are very similar to the ones obtained with the stationary TDF model. On the other hand, quantiles obtained with models including AMO are very different from the ones obtained with the stationary models. In general, the TDF model “Time + AMO” leads to very similar quantiles to the ones obtained with the TDF model “AMO”. The only exceptions are for the Quebec and Rimouski stations where the quantiles for the years 1974 and 1998 are distinct for these two models. These two stations are also the ones for which the TDF model “Time + AMO” obtains better goodness-of-fit statistics than the TDF model “AMO”. These last results can be explained by the fact that no important temporal trends are detected in the majority of time series while significant correlations are observed with AMO for the majority of durations for all stations.

## Discussion

This study proposes a nonstationary approach to the development of TDF curves. With this approach, parameters of the TDF relationship are conditional upon time-dependent covariates representing climate change and climate oscillation patterns (Climate variability). We found that the goodness-of-fit is improved when using a nonstationary approach with covariates related to climate variability. For most stations in the study area, the impacts of considering the temporal trend on quantiles are lower than the impacts of considering the climate index AMO. This illustrates the importance of introducing climate indices in nonstationary models. Future work may focus on the introduction of additional relevant low frequency climate oscillation indices in the nonstationary TDF model. For the study area considered in the present work, few significant trends are detected in the annual maximum temperature series. However, this may not be the case in other parts of the world where increasing heat wave frequency and intensity are reported^7,10,13^.

Nonstationarity in TDF curves, IDF curves or similar tools have not been introduced until recently^14,27^. The general procedure presented in this work can be applied to other variables such as precipitations (IDF curves) and floods (QDF curves) for which the influence of climate variability is relatively well documented^28–30^. The nonstationary TDF model can be useful in adaptive management where predictions of the evolution of extreme values are needed. This approach will allow integrating information concerning future extreme temperature characteristics in a number of applications such as the analysis of the safety of thermal and nuclear power plants. Indeed, the efficiency and safety of thermal and nuclear power plants are heavily affected by heatwaves by increasing the scarcity and temperature of cooling water and by reducing thermal efficiency^24^.

The approach proposed herein is useful if nonstationay models can be built for the near future. To achieve this, the covariate related to the temporal trend can be easily extrapolated. However, in the present approach, the climate index used is measured during the same period as the extreme temperature events (i.e. June-July-August). This is a limitation for prediction as the value of the climate index is unknown in the future. However, it is possible to predict climate indices^26,31^ and use these predictions in nonstationary TDF curves.

## Methods

### Nonstationary TDF relationship formulation

To build TDF curves, the time series of maximum average temperatures are derived from the daily maximum observed temperatures. For each year, the maximum average temperature value is extracted for a number of *D* durations *d*_*k*_, *k* = 1, …, *D*. The maximum average temperature for year *l*, denoted by *T*_*l*_(*d*), *l* = 1, …, *n*, is obtained using a moving average window during the summer season of June-August (JJA). In this formulation, *n* is the number of years with measurements and *d* ∈ [*d*_1_, *d*_*D*_].

The return level of *T*(*d*) for a return period of *R* years, denoted by *t*_*R*_(*d*), is given by:

1 $${t}_{R}(d)=\frac{a(R)}{b(d)}.$$

In this formulation, *t*_*R*_(*d*) has a separate functional dependence on the return period *R* and the duration *d*. The function *a*(*R*), determined by the distribution of *T*(*d*), defines curves that are parallel for different return periods *R*. The function *b*(*d*) models the shape of the TDF curves as a function of the duration *d* and can be expressed by:

2 $$b(d)={(d+\theta )}^{\eta },$$

where *θ* and *η* are the shape parameters subject to the inequality constrains that *θ* > 0 and 0 < *η* < 1.

If *F*_*T*(*d*)_ (*t*; *d*) denotes the distribution function of *T*(*d*), the scaled maximum average temperature *Y* = *T*(*d*) *b*(*d*) will also be distributed as *F*_*T*(*d*)_ (*t*; *d*) (i.e. ${F}_{T(d)}(t;d)={F}_{Y}({y}_{R})=1-\frac{1}{R}$). Since *a*(*R*) is the return level of *T*(*d*), the following expression is obtained:

3 $$a(R)={F}_{Y}^{-1}(1-\frac{1}{R}).$$

The Gumbel and the GEV are the probability distributions that are the most widely used to model climate extremes. The GEV is the limiting distribution for block maxima and includes the Gumbel distribution as a subset^32^. The GEV is used here to model *T*(*d*). The cumulative distribution function of the GEV is given by:

4 $$F(x)=\exp \{-\,{[1+\kappa (\frac{x-\mu }{\sigma })]}^{-1/\kappa }\},$$

where *μ*, *σ* and *κ* are the location, scale and shape parameters respectively. *F*(*x*) is defined for 1 + *κ*(*x* − *μ*)/*σ* > 0 where *σ* > 0. The general TDF relationship assuming that *T*(*d*) follows the GEV distribution is then given by the following expression:

5 $${t}_{R}(d)=\frac{a(R)}{b(d)}=\frac{\mu -\frac{\sigma }{\kappa }\{1-{[-\mathrm{log}(1-\frac{1}{R})]}^{-\kappa }\}}{{(d+\theta )}^{\eta }}.$$

In the nonstationary case, the distribution parameters are made dependent on covariates which can represent a climate oscillation index or time. The shape parameter of the GEV is usually kept constant in nonstationary analysis^33,34^. In this study, all shape parameters, including the parameter *κ*, *θ* and *η*, are also kept constant. Let us denote *U*_*l*_ and *V*_*l*_, the values during the *l*th year of two time-dependent covariates *U* and *V*. For non-stationary models with one covariate, the location parameter can be stationary or can depend linearly or quadratically on *U*_*l*_:

6 $${\mu }_{l}=\{\begin{array}{l}{\mu }_{0}\\ {\mu }_{0}+{\mu }_{1}{U}_{l}\\ {\mu }_{0}+{\mu }_{1}{U}_{l}+{\mu }_{2}{U}_{l}^{2}\end{array}$$

and the scale parameter can be stationary or can depend linearly on *U*_*l*_:

7 $${\sigma }_{l}=\{\begin{array}{l}{\sigma }_{0}\\ {\sigma }_{0}+{\sigma }_{1}{U}_{l}\end{array}.$$

For non-stationary models with two covariates, the location parameter can be stationary or can depend linearly or quadratically on *U*_*l*_ and *V*_*l*_:

8 $${\mu }_{l}=\{\begin{array}{l}{\mu }_{0}\\ {\mu }_{0}+{\mu }_{1}{U}_{l}+{\mu }_{2}{V}_{l}\\ {\mu }_{0}+{\mu }_{1}{U}_{l}+{\mu }_{2}{U}_{l}^{2}+{\mu }_{3}{V}_{l}\\ {\mu }_{0}+{\mu }_{1}{U}_{l}+{\mu }_{2}{V}_{l}+{\mu }_{3}{V}_{l}^{2}\\ {\mu }_{0}+{\mu }_{1}{U}_{l}+{\mu }_{2}{U}_{l}^{2}+{\mu }_{3}{V}_{l}+{\mu }_{4}{V}_{l}^{2}\end{array}\,$$

and the scale parameter can be stationary or can depend linearly on *U*_*l*_ and *V*_*l*_:

9 $${\sigma }_{l}=\{\begin{array}{l}{\sigma }_{0}\\ {\sigma }_{0}+{\sigma }_{1}{U}_{l}+{\sigma }_{2}{V}_{l}\end{array}.$$

The non-stationary models are built by considering any combinations of the models in equations (6) and (7) for one covariate and any combinations of the models in equations (8) and (9) for two covariates (the cases where both distribution parameters are constant are excluded since this is equivalent to the stationary model). For models with two covariates, it is not allowed for *μ*_*l*_(*σ*_*l*_) to depend only on one covariate and *σ*_*l*_(*μ*_*l*_) to depend only on the other covariate (see equations (8 and 9)). The relations of the distribution parameter *σ*_*l*_ with covariates are made constant or linear for the aim of obtaining simpler models considering that the number of parameters to be fitted increases rapidly with the complexity of the model, and that the size of the record may become a limiting factor. Parsimony considerations need to be taken into account in the selection of the optimum model to be adopted. The two covariates introduced in the nonstationary approach are the climate index AMO during summer and the year number denoted by Time. To obtain the AMO time series, the average of the AMO values over the months of the summer season (JJA) is computed. The covariate Time is defined by a series of integers incremented from 1 to the number of years of observed data.

### Maximum composite likelihood method

The vectors of the distribution parameters *ψ* = (*μ*, *σ*, *θ*, *η*) and *ψ* = (*μ*_0_, *μ*_1_, …, *σ*_0_, *σ*_1_, …, *κ*, *θ*, *η*) need to be estimated for stationary IDF curves and non-stationary IDF curves respectively. The method used here for the estimation of the parameters is the maximum composite likelihood. The characteristics and formulation of the nonstationary TDF model make it necessary to adopt this method instead of the classical maximum likelihood approach. Indeed, while observations from year to year are independent, the maximum temperatures over the different durations for the same year are dependent. Let us define *f*(*t*; *ψ*, *α*), the joint probability density of the random vector *T* = {*T*(*d*_1_), …, *T*(*d*_*D*_)} where *α* is a parameter vector that parameterizes the interdependence between temperatures corresponding to different durations and *ψ* parameterizes the marginal structure^35^. The full likelihood is then given by:

10 $$L(\psi ;\,t)=\prod _{l=1}^{n}f({t}_{1l},\,\ldots ,\,{t}_{Dl};\,\psi ,\,\alpha ),$$

where *t*_*kl*_ denotes the maximum average temperature for the year *l* and for the duration group *k*. However, the joint density *f*(*t*; *ψ*, *α*) is unknown, making the estimation of the full likelihood difficult. To overcome this difficulty, a simplified likelihood function for TDF curves is obtained by assuming the independence of the temperatures over the different durations^36,37^. This function is referred to as the independence likelihood^35^ and is given by:

11 $${L}_{ind}(\psi ;\,t)=\prod _{j=1}^{D}\,\prod _{l=1}^{n}\,f({t}_{jl};\,\psi ),$$

where *f*(*t*; *ψ*) represents the density function of *T*(*d*). The independence likelihood can be considered as the simplest case of a composite likelihood defined as an inference function derived by multiplying a set of component likelihoods^38^. Composite likelihood is used in several applications as surrogate for the full likelihood when it is too cumbersome or impractical to compute^39^. If *T*(*d*) is assumed to follow a GEV distribution, the probability function of *T*(*d*) is given by^37^:

12 $$T(d)\sim {\rm{GEV}}(\mu (d),\,\sigma (d),\,\kappa ).$$

In the non-stationary case, the distribution parameters *μ*(*d*) and *σ*(*d*) are expressed by:

13 $${\mu }_{l}(d)=\frac{{\mu }_{l}}{{(d+\theta )}^{\eta }},\,{\sigma }_{l}(d)=\frac{{\sigma }_{l}}{{(d+\theta )}^{\eta }}.$$

In practice, the log likelihood ${\ell }_{ind}(\psi ;\,t)=\,\mathrm{log}\,{L}_{ind}(\psi ;\,t)$ is maximized instead of *L*_*ind*_(*ψ*; *t*) with an optimization procedure. The optimization function *fmincon* in Matlab® is used to find $\hat{\psi }$, the estimate of *ψ* that maximizes ${\ell }_{ind}(\psi ;\,t)$. The algorithm used (interior-point) ensures that *σ*_*l*_ > 0: if *σ*_*l*_ < 0 for an iteration, the objective function returns a “not-a-number” value, the iterate is rejected and the next step is attempted.

For model comparison, information criteria such as the Akaike information criterion (AIC) or the Bayesian information criterion (BIC) are frequently used. Such criteria account for the goodness-of-fit and penalize more complex models. Let ${\rm{H}}(\psi )=-\,{\rm{E}}[{\nabla }^{2}{\ell }_{ind}(\psi ;\,T)]$ be the sensitivity or Hessian matrix and ${\rm{J}}(\psi )={\rm{Var}}\{\nabla {\ell }_{ind}(\psi ;\,T)\}$ be the variability matrix. Because of the assumption of independence among the likelihood terms in the definition of the independence likelihood, composite likelihood can be seen as a misspecified likelihood. In that case, the second Bartlett identity fails (i.e. H(*ψ*) ≠ J(*ψ*)) and classical criteria should be generalized. Analogous criteria for AIC and BIC based on composite likelihoods were introduced^39^ and have the following forms^38^:

14 $$\mathrm{CL} \mbox{-} \mathrm{AIC}=-\,2{\ell }_{ind}(\hat{\psi };\,t)+2{\rm{\dim }}(\psi ),$$

15 $$\mathrm{CL} \mbox{-} \mathrm{BIC}=-\,2{\ell }_{ind}(\hat{\psi };\,t)+{\rm{\dim }}(\psi )\,\mathrm{log}(n),$$

where dim(*ψ*) is the effective number of parameters estimated by tr{J(*ψ*)H(*ψ*)^−1^}. The sample estimate of the sensitivity matrix H can be obtained by:

16 $$\hat{{\rm{H}}}(\psi )=-\,\frac{1}{n}\sum _{l=1}^{n}\,\nabla u(\hat{\psi };\,{t}_{l}),$$

where $u(\psi ;\,{t}_{l})=\nabla {\ell }_{ind}(\psi ;\,{t}_{l})$ and *t*_*l*_ denotes the vector of maximum average temperatures for the year *l*. However, the cumbersome computation of the Hessians in equation (16) can be avoided. Given that the second Bartlett identity is valid for each individual likelihood term^38^, the matrix H can be obtained by the following sample estimate:

17 $$\hat{{\rm{H}}}(\psi )=\frac{1}{n}\,\sum _{k=1}^{D}\,\sum _{l=1}^{n}\,u(\hat{\psi };\,{t}_{kl})u{(\hat{\psi };{t}_{kl})}^{{\rm{T}}}.$$

The sample estimate of the variability matrix J can be obtained empirically by^38^:

18 $$\hat{{\rm{J}}}(\psi )=\frac{1}{n}\,\sum _{l=1}^{n}\,u(\hat{\psi };\,{t}_{l})u{(\hat{\psi };{t}_{l})}^{{\rm{T}}}.$$

## Electronic supplementary material

Supplementary information

## Data Availability

The maximum daily temperature data for Canada is freely available online from the Government of Canada: http://climate.weather.gc.ca/historical_data/search_historic_data_e.html. The monthly values of the climate index AMO are freely available online from the Earth System Research Laboratory of the National Oceanic and Atmospheric Administration: https://www.esrl.noaa.gov/psd/data/timeseries/AMO/.
