# Supplementary material for: Nonstationary Temperature-Duration-Frequency curves
Source: Sci Rep. 2018 Oct 19;8:15493. doi: 10.1038/s41598-018-33974-y (PMC6195567; doi:10.1038/s41598-018-33974-y)
Supplement: Supplementary file 1 — Supplementary information [file 41598_2018_33974_MOESM1_ESM.pdf]

Supplementary information for

Nonstationary Temperature-Duration-Frequency curves

Taha B.M.J. Ouarda[1,*] and Christian Charron[1]

[1]Canada Research Chair in Statistical Hydro-Climatology, INRS-ETE, 490 de la Couronne, Québec, QC, G1K 9A9, Canada

*Corresponding author:

Email: taha.ouarda@ete.inrs.ca

Tel: +1 418-654-3842

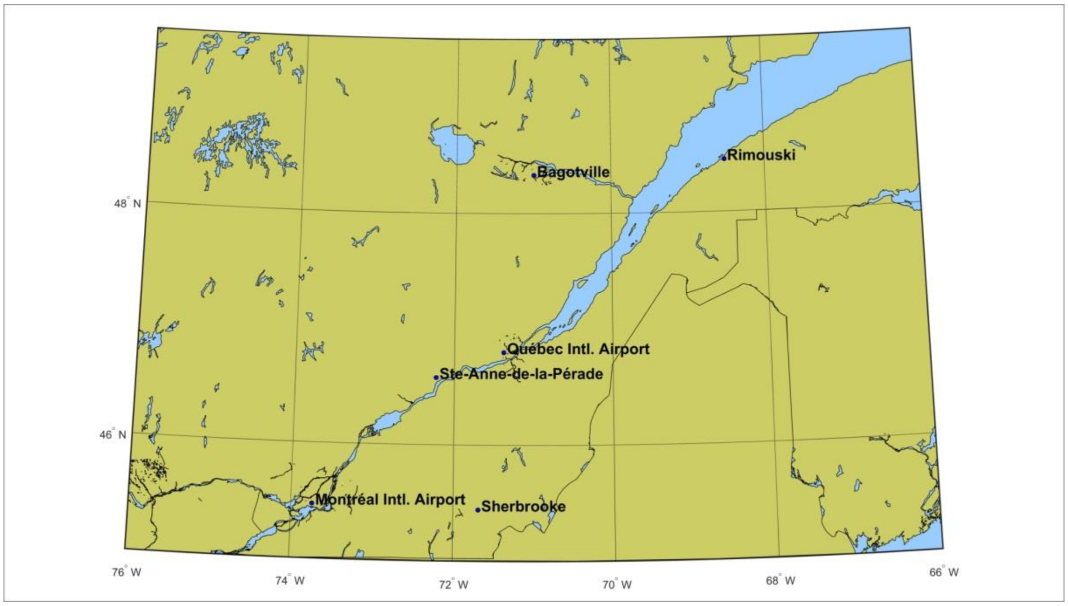

Supplementary Figure 1. Geographical locations of the selected stations.

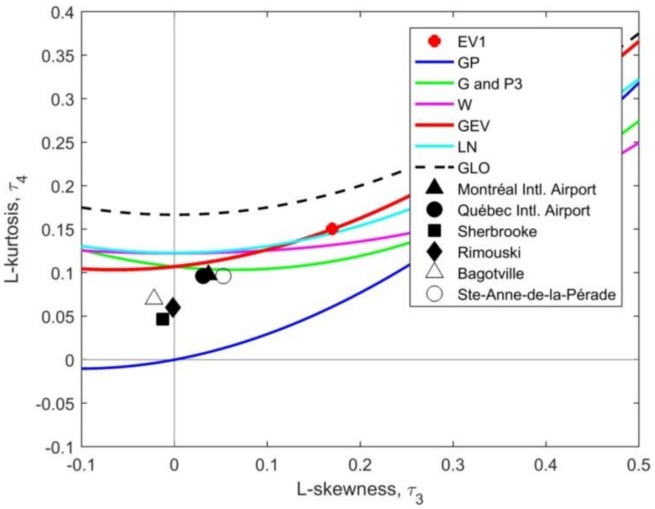

Supplementary Figure 2. L-moment ratio diagram and sample L-moments of the selected stations.

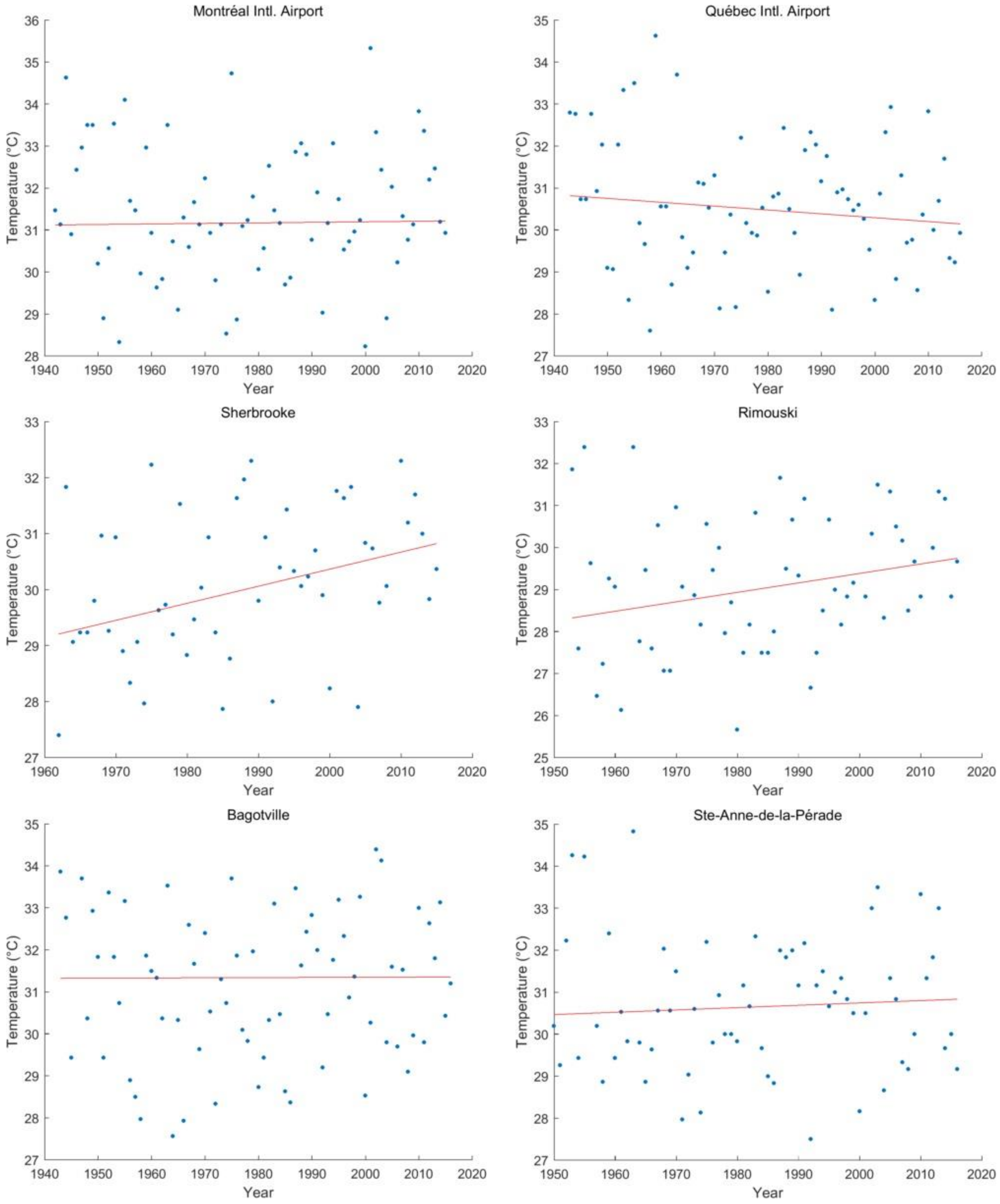

Supplementary Figure 3. Annual maximum temperatures for 3-day duration.

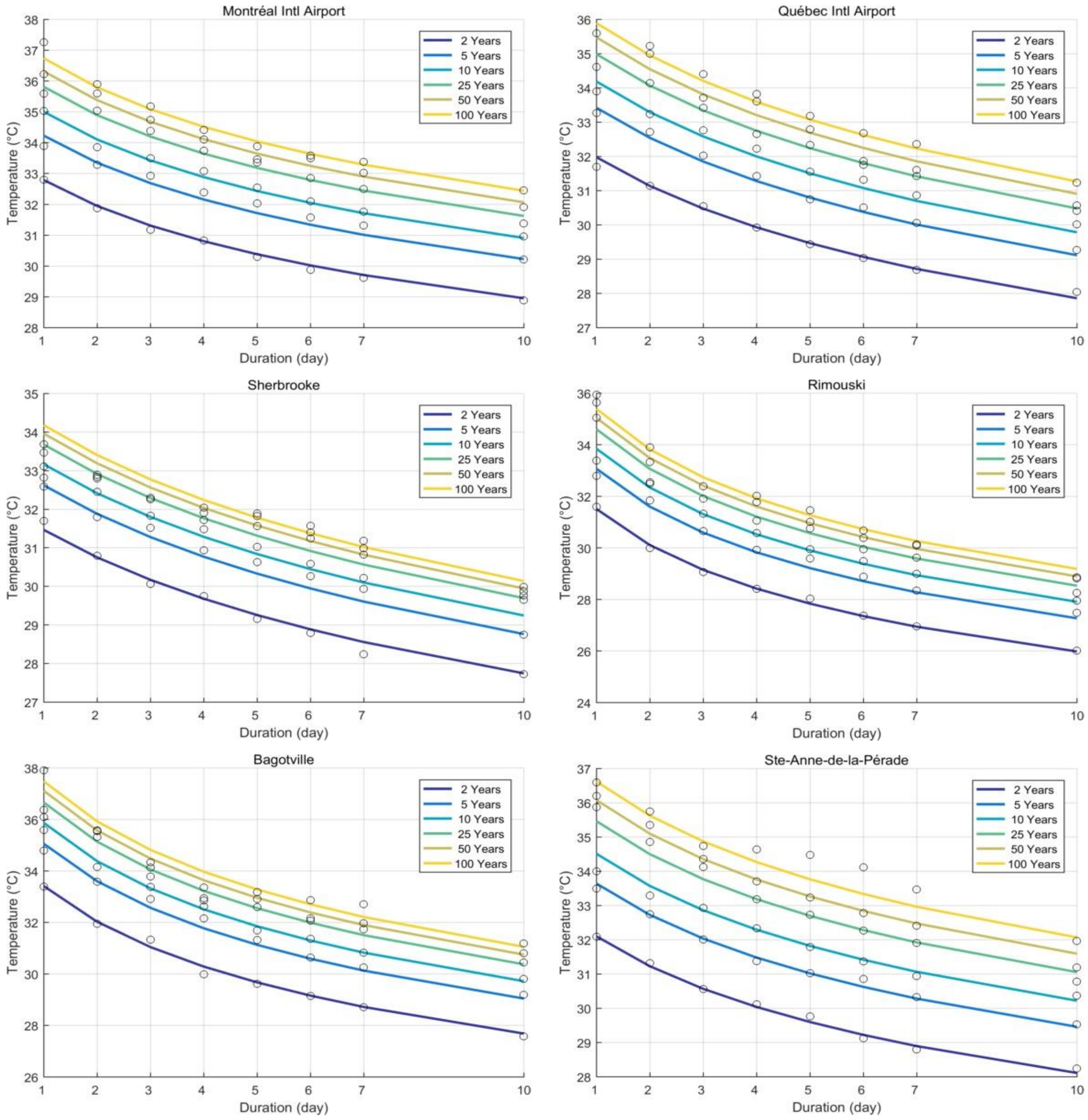

Supplementary Figure 4. TDF curves for the stationary case.

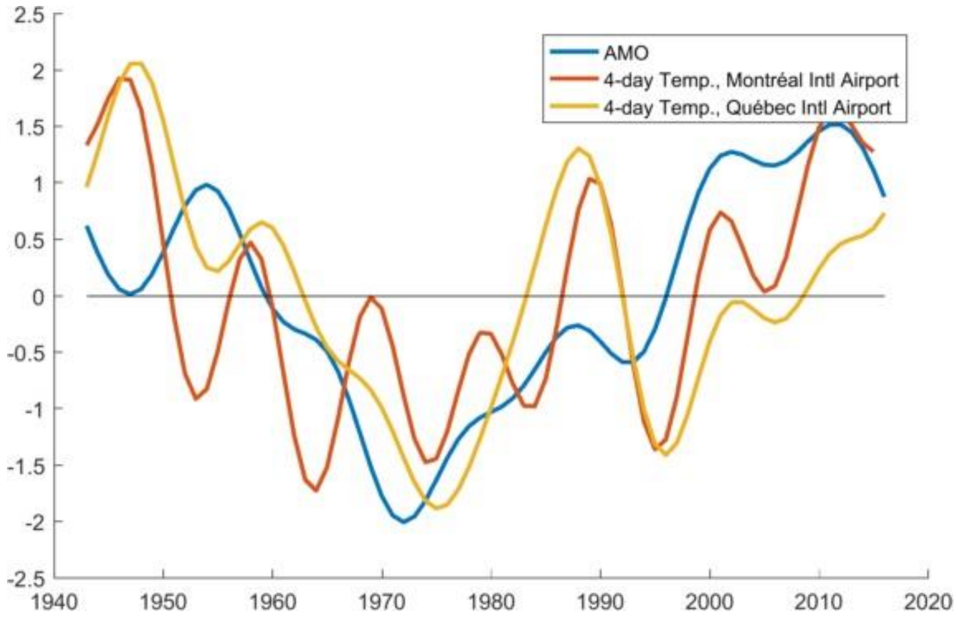

Supplementary Figure 5. Filtered AMO for the summer season and 4-day maximum temperature for the Montréal and Quebec Intl. Airport stations. Time series were low-pass filtered with the Lanczos method.

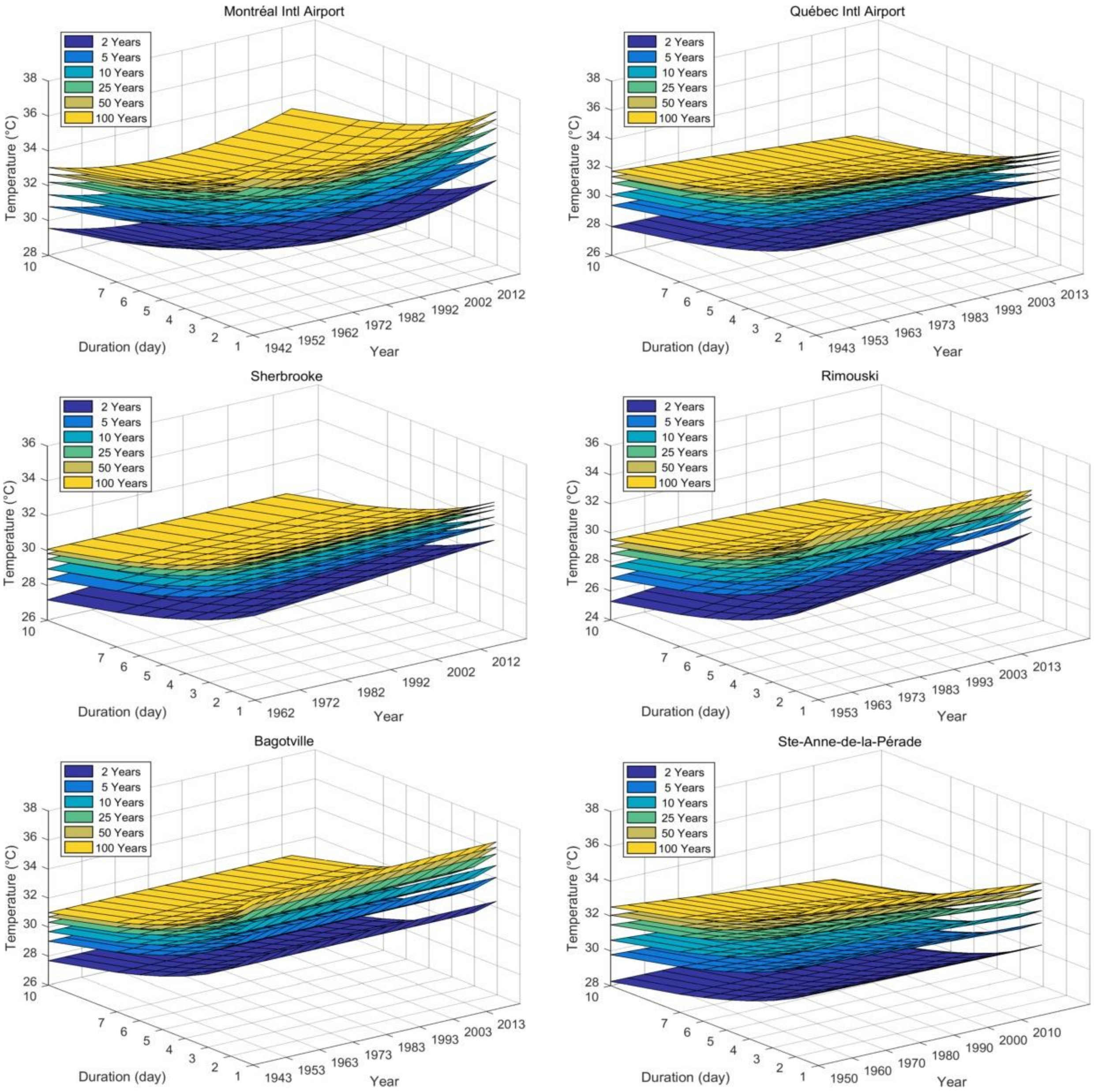

Supplementary Figure 6. Nonstationary TDF surfaces with Time as covariate.

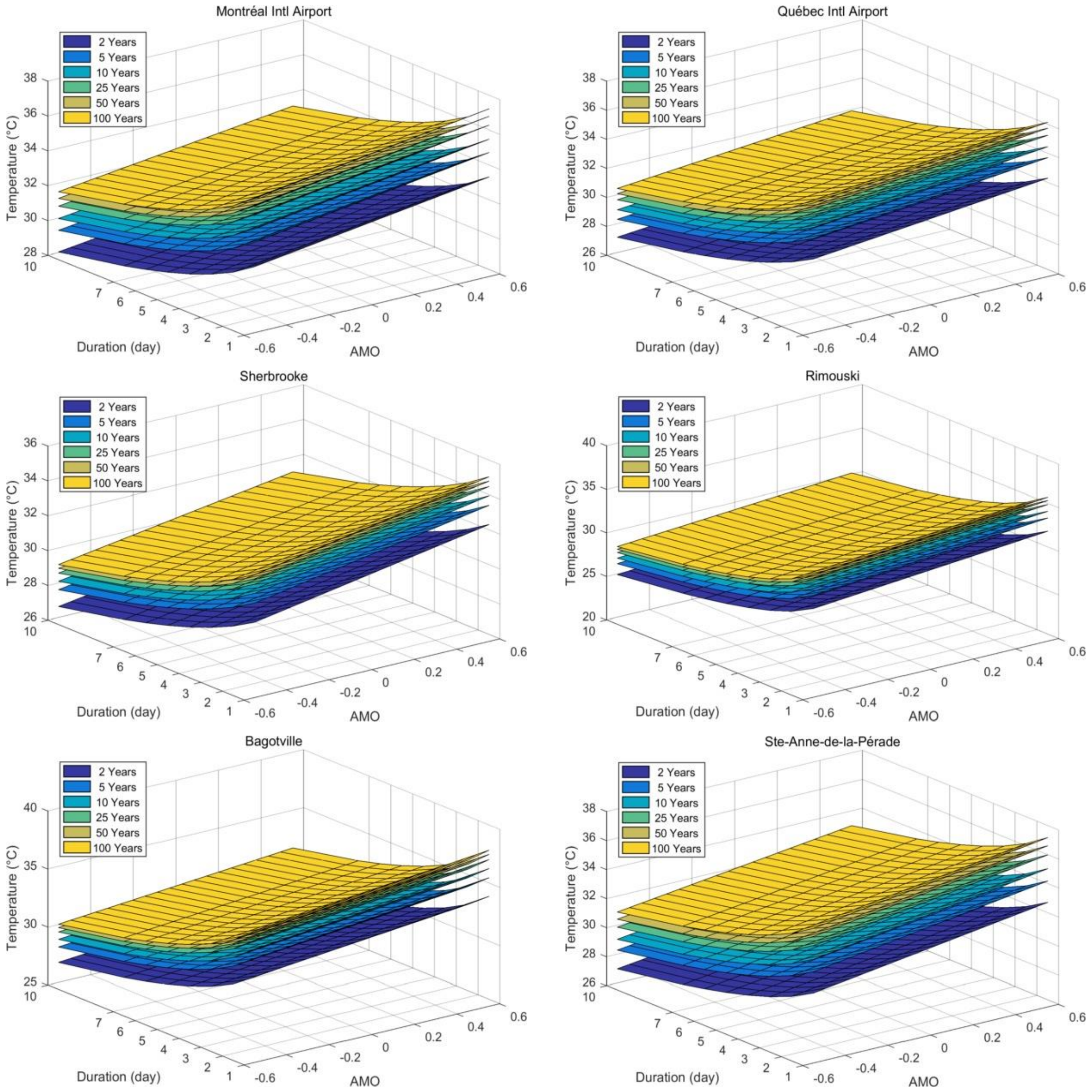

Supplementary Figure 7. Nonstationary TDF surfaces with AMO as covariate.

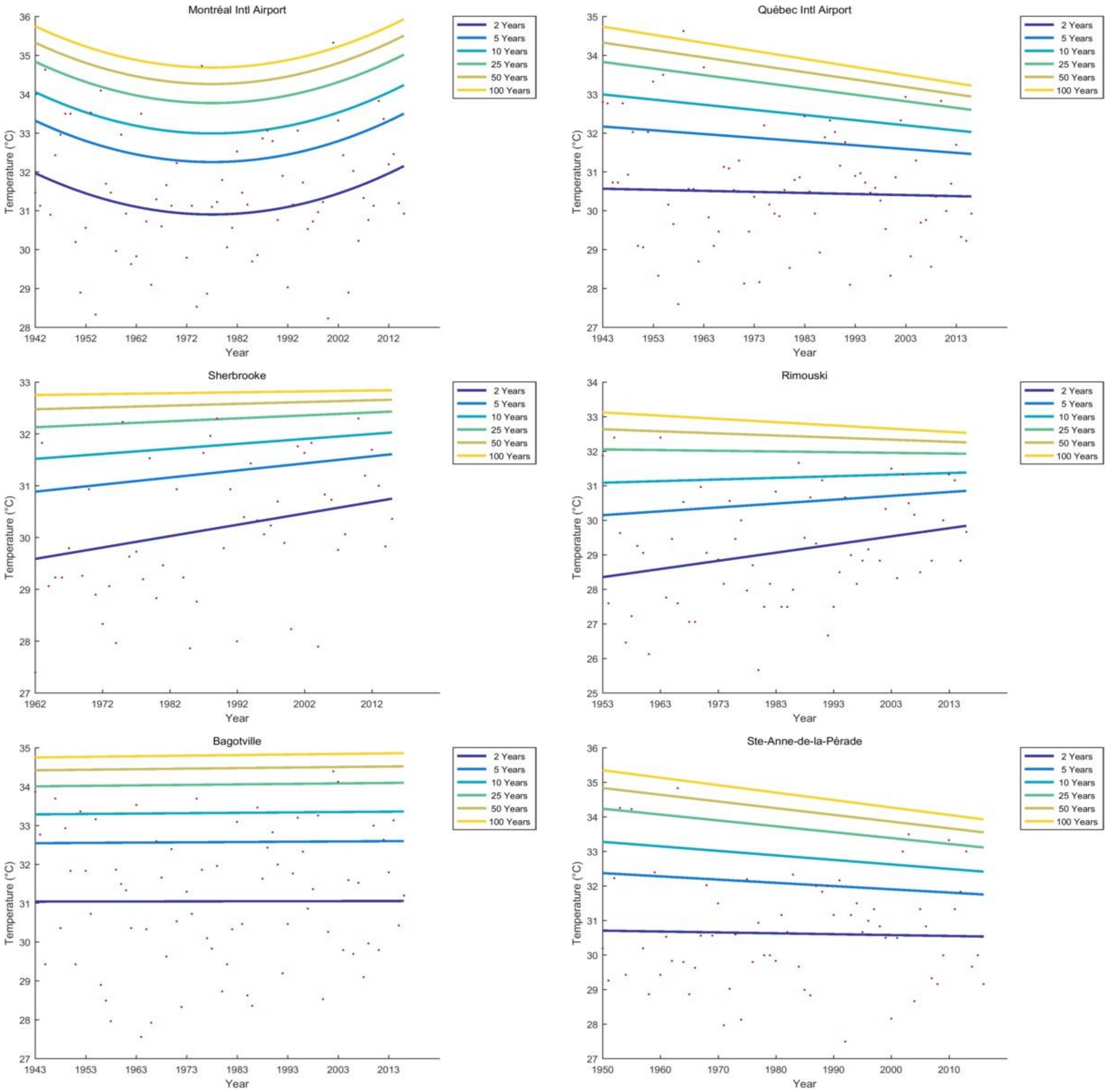

Supplementary Figure 8. 3-day maximum temperature against the year for the nonstationary TDF model with Time as covariate.

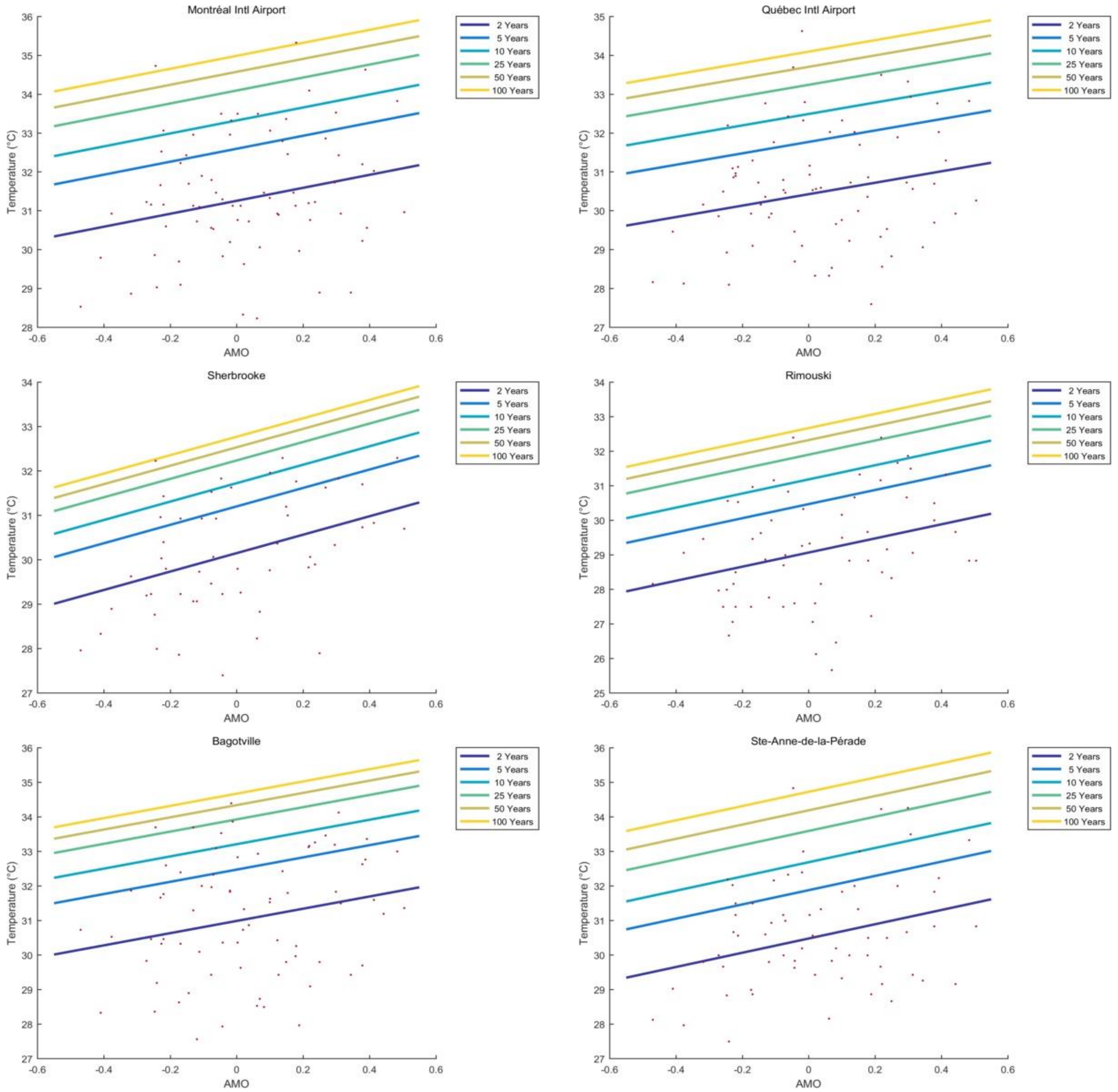

Supplementary Figure 9. 3-day fixed duration maximum temperature against AMO for the nonstationary TDF model with AMO as covariate.

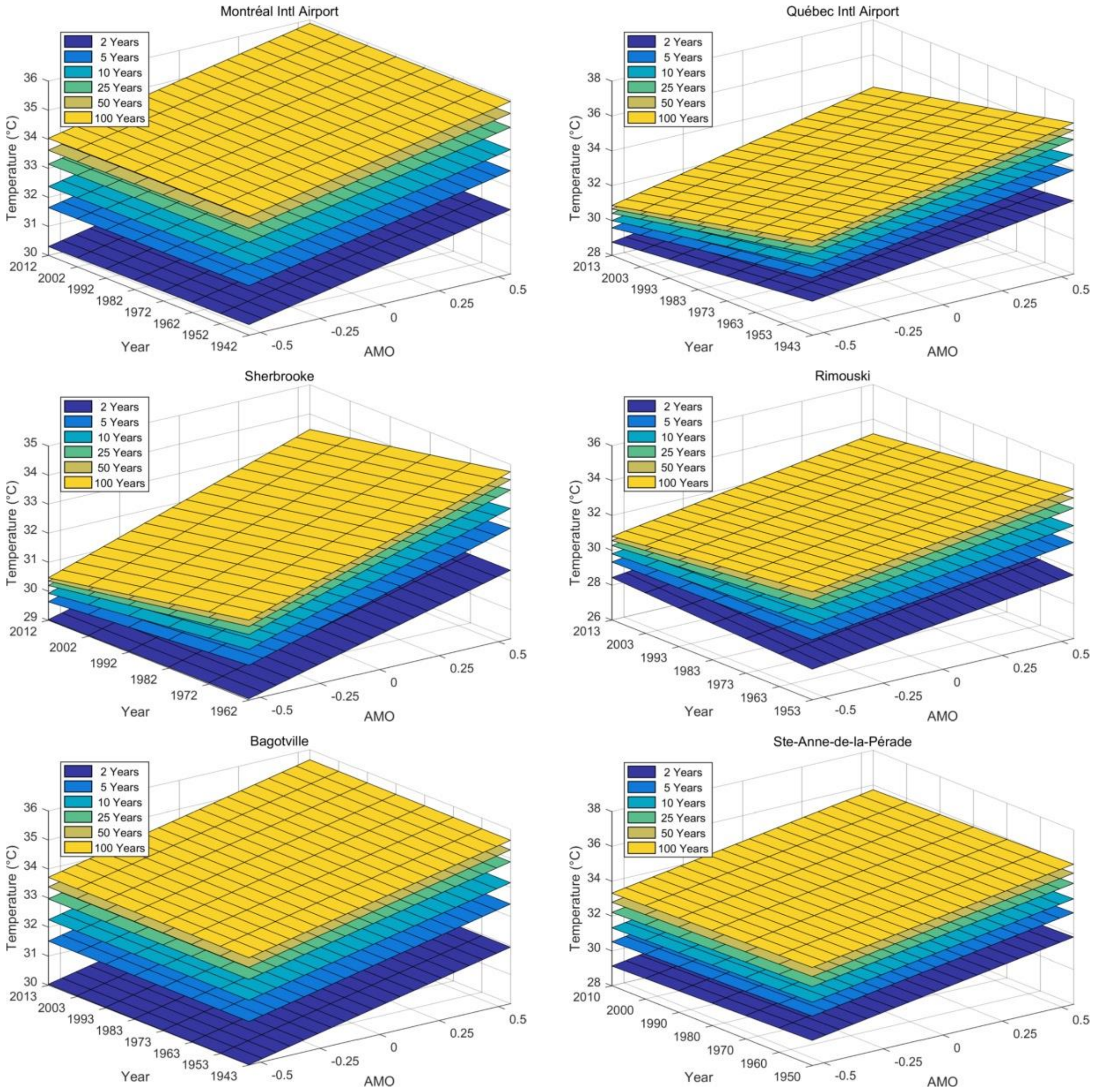

Supplementary Figure 10. Nonstationary TDF surfaces with AMO and Time as covariates for a 3-day fixed duration.

Supplementary Table S1. Slopes (°C/year) and Mann-Kendall trend test conclusions for the annual maximum temperatures.

Duration	Station					
	Montréal Intl. Airport	Quebec Intl. Airport	Sherbrooke	Rimouski	Bagotville	Ste-Anne-de-la-Pérade
1	0.000	-0.009	0.011	0.015	0.000	0.000
2	0.002	-0.013	**0.022**	0.016	0.005	0.005
3	0.001	-0.009	**0.030**	**0.023**	0.000	0.006
4	0.004	-0.009	**0.036**	**0.028**	0.002	0.009
5	0.008	-0.011	**0.031**	**0.030**	0.001	0.010
6	0.007	-0.010	**0.025**	**0.027**	0.001	0.009
7	0.005	-0.007	**0.023**	**0.027**	0.003	0.010
10	0.004	-0.005	**0.023**	**0.022**	0.007	0.006

Bold character denotes significant correlation at a significance level of 10% using the Mann-Kendall test.

Supplementary Table 2. Correlations between the annual maximal temperature for selected durations and AMO during the summer season (JJA).

Station	Duration			
	1	3	5	7
Montréal Intl. Airport	0.220	**0.233**	**0.252**	**0.287**
Quebec Intl. Airport	0.217	0.193	**0.236**	**0.278**
Sherbrooke	0.292	**0.431**	**0.387**	**0.379**
Rimouski	**0.183**	**0.319**	**0.331**	**0.298**
Bagotville	0.099	0.223	**0.258**	**0.264**
Ste-Anne-de-la-Pérade	**0.287**	**0.285**	**0.309**	**0.344**

Bold character denotes significant correlation at significance level of 5%.
